# Artery segmentation and atherosclerotic plaque quantification using AI for murine whole slide images stained with oil red O

**DOI:** 10.1038/s41598-025-93967-6

**Published:** 2025-04-23

**Authors:** Johann Christopher Engster, Tobias Reinberger, Nele Blum, Pascal Stagge, Thorsten M. Buzug, Zouhair Aherrahrou, Maik Stille

**Affiliations:** 1 Fraunhofer IMTE, Fraunhofer Research Institution for Individualized and Cell-Based Medical Engineering, 23562 Lübeck, Germany; 2 Institute for Cardiogenetics, University of Lübeck, 23562 Lübeck, Germany; 3 Institute of Medical Engineering, University of Lübeck, 23562 Lübeck, Germany; 4 DZHK (German Centre for Cardiovascular Research), Partner Site Hamburg/Kiel/Lübeck/Greifswald, 23562 Lübeck, Germany; 5 University Heart Center Lübeck, 23562 Lübeck, Germany

**Keywords:** Artery segmentation, Ensemble, Machine learning, Oil red O, Plaque segmentation, Bioinformatics, Imaging, Software, Cardiovascular biology, Cardiovascular genetics

## Abstract

Atherosclerosis is the leading cause of death in Western industrial nations. To study the etiology of plaque progression, atherosclerotic mouse models are widely used. Traditionally, analyzing the obtained histological whole slide images of Oil Red O-stained aortic roots required manual segmentation. To accelerate this process, an artificial intelligence-driven solution is proposed that comprises three stages: (1) defining the region of interest (ROI) of the aortic root using a YOLOv8l object detector, (2) applying supervised machine learning with ensembles of U-Net++ networks for artery segmentation using ROI masks, and (3) performing plaque segmentation within arterial walls with the unsupervised W-Net method. To establish a robust segmentation pipeline, we benchmark our methods using manually created masks ($\text {n}=6085$ for artery segmentation, $\text {n}=1089$ for plaque segmentation). A key finding is that an ensemble of U-Net++ networks applied on ROI masks outperformed single network architectures. Through a novel combination strategy, the ensemble output can be easily modified, paving the way for a quick and robust application in the lab. Finally, a case study utilizing published mouse data ($\text {n}=373$ slices) underscored the ability of our optimized pipeline to replicate human-made plaque predictions with a high correlation (Pearson’s $\text {r}=0.91$) and reproduce biological insights derived from manual analysis.

## Introduction

Caused by the modern Western lifestyle and risk factors such as hypertension or obesity, the prevalence of coronary artery disease (CAD) is rising, thereby dramatically reducing the life expectancy in industrial nations^1^. The main reason for CAD is atherosclerosis, a multifactorial disease of the cardiovascular system^2,3^, in which so-called plaques form in the arteries. These can lead to follow-up diseases like ischemic heart disease, thrombosis, or strokes^2,4^.

To investigate atherosclerosis and treatment options, mice are the most frequently used animal model. The two common modes for quantifying the severity of atherosclerosis in mice are *en-face* analyses of the aorta and cross-sections of the aortic root^5^. Lesions can be visualized in cross sections using hematoxylin and eosin (H&E) staining in combination with Oil Red O (ORO) staining. The latter is widely adopted in multiple publications to visualize lesions as lipid-laden macrophages are considered the major cell type in lesions^6–9^. Mice lacking the Apolipoprotein E gene (*Apoe*-knockout) were fed with a high-fat diet for several weeks and dissected to analyze plaque formation in the arteries, following a precise dissection protocol by Centa et al.^10^. Subsequently, Whole Slide Images (WSIs) of the aortic root were created and stained with ORO to highlight plaque build-up^11^. The arteries inside those WSIs were manually segmented by trained technicians, making it a very time-consuming and expensive task. Regarding plaque quantification, a threshold-based technique was applied for the studies presented by Achner et al.^7^, which required manual parameter inputs.

**Fig. 1:**
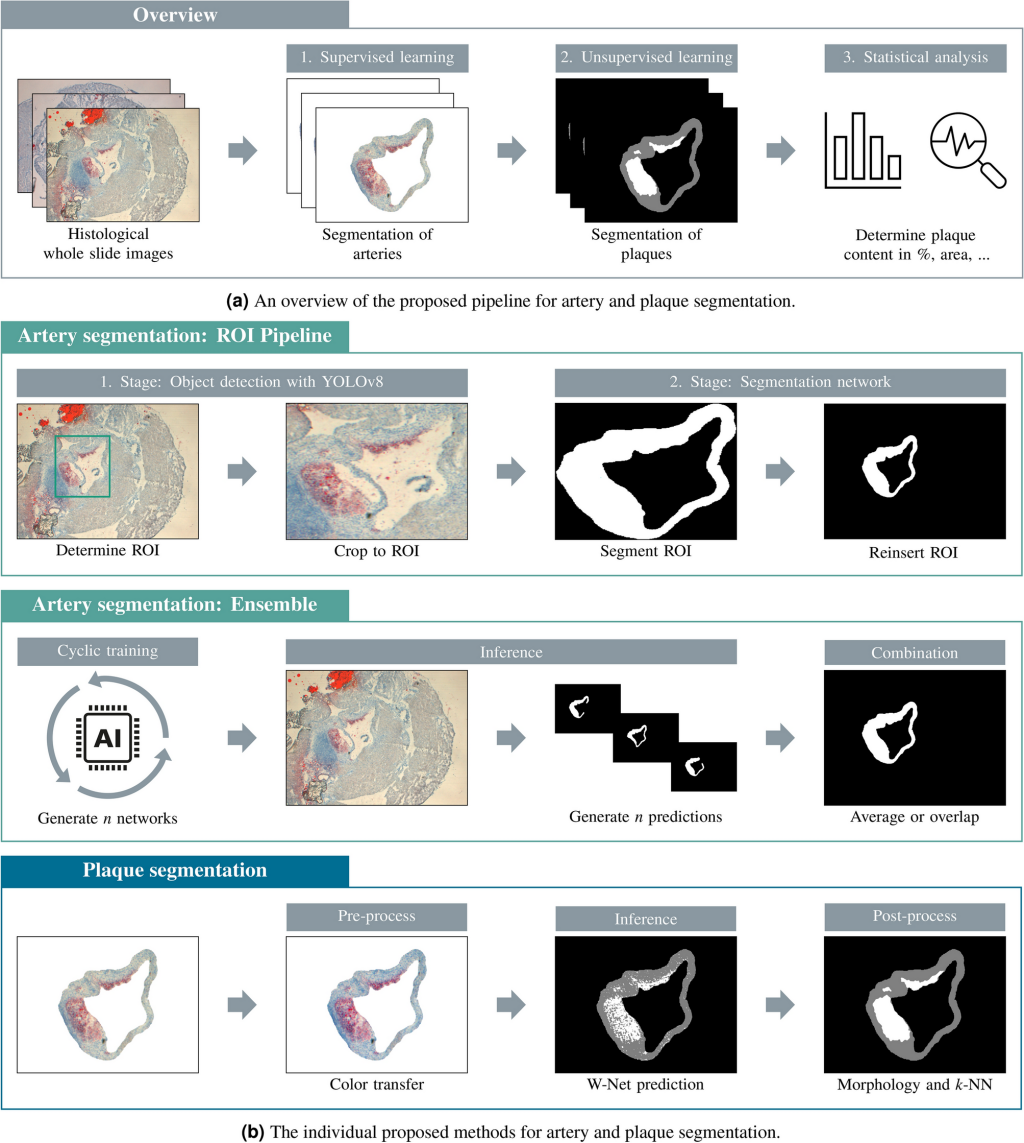
(**a**) Artery and plaque segmentation combines supervised and unsupervised learning methods for automatic lesion characterization. Initially, input WSIs are inserted into a segmentation network, which was trained in a supervised manner on a large hand-labeled dataset. This yields a binary segmentation mask of the artery area. The (cropped) arterial region is then processed by the W-Net, an unsupervised network trained on a selected dataset for analysis purposes. After post-processing, both the artery and plaque regions can be used for statistical analysis. (**b**) Individual steps for artery and plaque segmentation in detail. The upper and middle panel illustrates the region of interest (ROI) pipeline and the ensemble structure for artery segmentation. The lower panel illustrates the proposed strategy for plaque segmentation.

However, the rise of artificial intelligence (AI) has paved the way for rapid and automatic quantification of lesion characteristics. Despite the development of numerous segmentation approaches in recent years, multiple challenges remain for artificial intelligence (AI) applications in pathology. These include limited training data, high resolutions of WSIs, differences in the staining protocol, or variation in microscopic settings, including different types of microscopes^12,13^.

State-of-the-art supervised segmentation methods utilize either a single network architecture^14^ or a combination of neural networks (pipelines^15^ and ensembles^12,16–19^) to overcome these challenges. Network ensembles can surpass the performance of a single network as they can overcome local optima^16^. For unsupervised segmentation methods without any ground truth (GT) at hand, different approaches using deep learning (DL)^20^ and classical computer vision (CV)^21,22^ are applied for WSI segmentation.

In this work, both supervised and unsupervised learning for segmentation tasks on WSIs of arteries with atherosclerotic lesions have been investigated using various network architectures. Our findings demonstrate that task-specific ensembles outperform single networks, providing reliable artery segmentation. Additionally, the unsupervised network architecture correctly detects the plaques inside the arteries. A case study performed on ORO-stained WSIs from *Cyp17a1* deficient mice by Aherrahrou et al.^9^ shows that the entire analysis can be automated with near-human performance, providing insights into atherosclerotic developments.

## Methods

The proposed pipeline for automatic AI-assisted lesion characterization comprises three main stages, as shown in Fig. 1a. First, the arterial regions within the WSIs are determined. Second, the plaque regions inside these arterial regions are estimated. Thirdly, a statistical analysis is conducted, which combines the results of the two previous stages.

### Artery segmentation

For artery segmentation, various supervised machine learning (ML) methods have been proposed. During the training process, the network’s predictions are compared to the corresponding label, with a loss function used to quantify the difference between them. The network weights are then updated to decrease the loss function. As a baseline, an individual segmentation network was fine-tuned and trained. Additionally, a network pipeline and ensembles were investigated to enhance the final output mask and minimize overhead for technicians, as incorrectly segmented arteries would require major revisions.

For the individual network, several segmentation architectures were trained and compared. Specifically, the U-Net^23^, the U-Net++^24^, the feature pyramid network (FPN)^25^, and pyramid attention network (PAN)^26^ architectures were tested. These state-of-the-art segmentation networks have widespread applications in the medical, industrial, and research sectors. For implementation, the Segmentation Models PyTorch (SMP)^27^ library was utilized, offering a variety of pre-build architectures, encoders, loss functions, and metrics for the PyTorch^28^ machine learning framework. All networks were trained for 100 epochs with a batch size of 16 using the ADAM optimizer^29^ on four NVIDIA RTX A6000 GPUs with 48 GB of memory each. The WSIs were resized to a resolution of $640\times 480$ pixels to maintain the original aspect ratio, which was 4:3 for all images. Early stopping was employed to terminate the training when no improvement occurred over 10 epochs. Different encoders from the ResNet^30^ and ResNext^31^ families were investigated, with learning rates ranging from $1\cdot 10^{-2}$ to $1\cdot 10^{-5}$. Further, the effects of basic data augmentations (horizontal & vertical flips, and rotations) and advanced augmentations (color jittering and deformations), were also examined. Lastly, different distribution-based and region-based loss functions^32^ were applied to optimize segmentation. Preliminary studies showed that the ResNeXt-101 (32 $\times$ 16d)^31^ encoder, with a learning rate of $1\cdot 10^{-4}$ and a combined Jaccard, Tversky ($\alpha =0.3, \beta =0.7$)^33^ and Binary Cross Entropy (BCE) loss^32^ performed best.

To capture the artery in utmost detail, being surrounded by background tissue structures, a region of interest (ROI) pipeline was used (see Fig. 1b). In the first stage, the ROI of the artery was predicted by an object detection network. In the second stage, the high-resolution WSI was cropped to this specific ROI and passed into a segmentation network. For ROI generation, the state-of-the-art You Only Look Once (YOLO) object detector^34^ was used. YOLO divides the input image into a grid structure and predicts bounding boxes and classes for each cell. We used the newest version, YOLOv8^35^, which offers a wide range of pre-designed model architectures. For our application, the YOLOv8l (large) model was selected.

Lastly, an ensemble method combining multiple networks was applied to improve the robustness of arterial segmentation (Fig. 1b). The individual output segmentation masks of a single WSI were combined into one high-precision mask. To obtain different networks, an explicit ensemble technique called snapshot ensemble, introduced by Huang et al.^36^, was used. During a single training process, multiple different local network optima were achieved through a cyclic learning rate^16,36^ using the PyTorch cyclic learning rate scheduler. Hence, the ADAM optimizer was replaced by the stochastic gradient descent (SGD) optimizer with a momentum of 0.9. The step size of the scheduler was set to the number of batches per epoch times five, allowing the learning rate to reach its maximum in five epochs and then decrease to its minimum in another five epochs. The base learning rate was set to $1\cdot 10^{-4}$ and increased by a factor of $10^3$ at its maximum. After 100 training epochs, the ten different local optima were combined into an ensemble. For the ensemble of predicted segmentation masks, two different combination strategies were evaluated. In the first approach, the network outputs were averaged before being passed through the final output function^16^. In the second approach, an adaptation of majority voting was used, which can be interpreted as an overlapping of the different *n* output masks. Moreover, an added hyperparameter $\alpha$ can be used to fine-tune the trade-off between false positive (FP) and false negative (FN) predictions. Thus, each pixel was classified as part of the artery only if $\alpha \ge n$ output masks agreed on that pixel. With a higher $\alpha$, more network outputs have to agree on the pixel’s classification, reducing the number of FPs. This approach can be efficiently formulated with matrix notation for fast application after individual output masks have been calculated, making the final output mask more independent of individual network errors.

### Plaque segmentation

Once the artery segmentation was completed, the next step involved segmenting the plaque regions. Since no GT was available for this task, an unsupervised segmentation approach was utilized (see Fig. 1b). This approach employed a modified version of the unsupervised W-Net^37^ architecture, followed by post-processing techniques to minimize artifacts.

The W-Net architecture, introduced by Xia and Kulis^37^, combines two U-Nets for unsupervised segmentation and is based on the principle of an autoencoder. The network’s input is passed through an encoder, which generates a latent space representation. This representation is then reconstructed by a decoder, aiming to closely match the original image. Consequently, the encoder and decoder weights were updated through a reconstruction loss. Additionally, to achieve distinct class assignments in the latent space, a soft-n-cut loss was used to update the encoder weights. After the training, the encoder weights were used to generate the segmentation output mask. Task-specific weightings were applied to increase the W-Net’s performance for plaque segmentation^38^. While color deviations did not significantly affect the performance of the artery segmentation network, the W-Net encountered difficulties with inhomogeneous input images. To address this, a color transfer technique^39^ was applied during inference. This method allows the color space of one image to be transferred to a target image. In our application, the color space from the whole target dataset was transferred to all target images, resulting in a homogeneous color space across the whole dataset. To extract information from only one class (i.e., ORO-stained plaques), we employed a post-processing approach that included morphological operations implemented in OpenCV^40^ and the k-nearest neighbors (*k*-nn)-algorithm. This replaces the post-processing described by the original W-Net^37^ authors, which consists of conditional random fields and hierarchical segmentation of several classes.

### Datasets

The artery segmentation networks were trained on a large in-house dataset consisting of 6085 WSIs originating from 530 different mice collected over a time span of ten years. This dataset included 13 diverse subdatasets, covering a high range of resolutions (from $960 \times 720$ up to $2720 \times 2048$ pixels). Each subdataset corresponded to one whole dissection session, where various mice strains were examined. In total, different feeding procedures, further knockout genes, and environmental factors were investigated. The data was split 60/20/20 for Training/Validation/Testing.

For plaque segmentation, a separate dataset was selected due to its availability of a hand-labeled reference (HLR) for comparative analysis. Typically, evaluating unsupervised methods quantitatively is challenging, however, this reference enabled the calculation of various metrics. In total, 1089 WSIs from 103 different mice with hand-labeled plaque regions were available.

Finally, a performance test of the whole pipeline - including artery segmentation, plaque segmentation, and a comparison of the statistical analysis - was conducted in a case study involving *Cyp17a1* deficient mice, which are prone to a higher development of atherosclerotic lesions. The dataset of that case study consisted of WSIs from a recently published mice study^9^ that examined atherosclerotic lesions in two transgenic mouse strains ($\textit{Cyp17a1}^{-/-} \times \textit{Apoe}^{-/-}$ and $\textit{Cyp17a1}^{+/+} \times \textit{Apoe}^{-/-}$) under a western-type diet (WTD) or chow diet as control (see Aherrahrou et al.^9^ for more details). After filtering, the dataset included nearly 373 WSIs from 48 different mice. For each WSI, a hand-labeled artery mask and a threshold-based reference (TBR) for the plaque amount inside the arterial region were available. Compared to the original publication, some WSIs from mice were discarded because they were saved in a non-lossless JPG format.

### Ethics statement

All animal experiments were approved by the German animal studies committee of Schleswig-Holstein, and animals were maintained and handled according to international guidelines and all methods were carried out in accordance with relevant guidelines and regulations.

### Performance test of segmentation

The evaluation of segmentation approaches primarily relied on the mean Intersection over Union (mIoU), a common metric for segmentation tasks. Hand-selected masks were defined as ground truth (GT) and compared to network predictions using the following formula:

$$\begin{aligned} \text {mIoU} = \frac{\text {TP}}{\text {TP}+\text {FN}+\text {FP}}, \end{aligned}$$

where TP are true positives, FP are false positives, and FN are false negatives. We categorized the IoU of the masks into three groups: (1) low-quality masks with $\text {mIoU} \le 0.5$, which would require major revisions; (2) medium-quality masks with $0.5< \text {mIoU} \le 0.7$, which might require fine-tuning; and (3) high-quality masks with $\text {mIoU} > 0.7$ suitable for further analyses without fine-tuning. It is important to note that manual segmentation by the technicians is subjective, and even a second human annotation will not achieve a 1.0 mIoU. Thus, small deviations from the GT were expected and should not interfere with plaque estimation. To quantify the rates of FP and FN, precision and recall were also used for the evaluation, respectively.

## Results

### Artery segmentation

In this section, we evaluate and compare the three main artery segmentation methods. Table 1 (Test set) shows the scores of the quantitative evaluation on the test dataset. Out of all individual networks, U-Net++ performed the best, followed by the U-Net, the PAN, and the FPN. Therefore, the U-Net++ network was defined as the baseline for further analyses of pipelines and network ensembles. To achieve higher precision in artery segmentation, a ROI pipeline was employed as a pre-processing step. This pipeline combined an object detector to propose a bounding box of the arterial region with a segmentation network. Using the ROI defined by YOLOv8l as input for the U-Net++ resulted in better performance when being compared to baseline (mIoU$=0.829\pm 0.065$ vs. mIoU$=0.826\pm 0.064$), which was comparable to the manual GT-ROI selection. However, the number of masks with a low mIoU slightly increased with both methods. Next, we evaluated the snapshot ensemble. Output masks from the different networks were combined into a high-precision mask using two strategies: overlapping with varying $\alpha$ or mask averaging. All combination strategies almost equally outperformed the baseline U-Net++ and performed similarly to the ROI pipeline. Increasing the hyperparameter $\alpha$ increased precision at the expense of recall. Finally, both the ROI pre-processing and the snapshot ensemble were combined. Therefore, an ensemble was trained using ROI masks as input. The combined ROI ensemble used half of the regular ensemble and half of the ROI ensemble weights. This approach helped to overrule potential false negatives (FN) or false positives (FP) resulting from faulty bounding boxes. This method further increased segmentation performance to a mIoU of $0.833\pm 0.062$, yielding more than $97\%$ high-quality masks ($\text {mIoU}>0.7$). Further, $2.4\%$ of the images remained in the medium range and only 0.5% were of low quality.

**Table 1:** Performances of several segmentation approaches and datasets.

	Method	Precision	Recall	mIoU	mIoU in % low / medium / high
Test set	Individual network				
	FPN	$0.885\pm 0.060$	$0.919\pm 0.054$	$0.820\pm 0.066$	0.4 / 4.1 / 95.5
	PAN	$0.900\pm 0.053$	$0.909\pm 0.055$	$0.825\pm 0.065$	0.5 / 3.5 / 96.0
	U-Net	$0.897\pm 0.056$	$0.913\pm 0.055$	$0.826\pm 0.066$	0.5 / 3.5 / 96.0
	U-Net++	$0.891\pm 0.056$	$0.919\pm 0.053$	$0.826\pm 0.064$	0.4 / 3.5 / 96.1
	ROI pipeline (U-Net++)				
	YOLOv8l	$0.899\pm 0.054$	$0.914\pm 0.056$	$0.829\pm 0.065$	0.6 / 3.0 / 96.5
	GT	$0.901\pm 0.049$	$0.915\pm 0.055$	$0.831\pm 0.062$	0.5 / 2.6 / 96.9
	Snapshot ensemble (U-Net++)				
	$\alpha =4$	$0.884\pm 0.058$	$0.933\pm 0.048$	$0.830\pm 0.063$	0.6 / 3.0 / 96.5
	$\alpha =6$	$0.897\pm 0.054$	$0.920\pm 0.052$	$0.832\pm 0.062$	0.4 / 3.0 / 96.6
	$\alpha =8$	$0.910\pm 0.050$	$0.905\pm 0.056$	$0.830\pm 0.063$	$\mathbf {0.3}$ / 3.4 / 96.2
	Average	$0.895\pm 0.055$	$0.922\pm 0.051$	$0.832\pm 0.062$	0.4 / 3.0 / 96.6
	Combined ROI ensemble (U-Net++)				
	$\alpha =4$	$0.882\pm 0.058$	$\mathbf {0.935\pm 0.048}$	$0.831\pm 0.063$	0.5 / 2.7 / 96.8
	$\alpha =6$	$0.900\pm 0.052$	$0.919\pm 0.052$	$\mathbf {0.833\pm 0.062}$	0.5 / $\mathbf {2.4}$ / $\mathbf {97.1}$
	$\alpha =8$	$\mathbf {0.915\pm 0.047}$	$0.900\pm 0.057$	$0.830\pm 0.062$	0.5 / 3.0 / 96.5
	Average	$0.897\pm 0.053$	$0.922\pm 0.051$	$\mathbf {0.833\pm 0.062}$	0.5 / 2.5 / 97.0
Case study	Individual network				
	U-Net++	$0.828\pm 0.081$	$0.906\pm 0.101$	$0.758\pm 0.090$	1.6 / 17.4 / 81.0
	Combined ROI ensemble (U-Net++)				
	$\alpha =4$	$0.827\pm 0.078$	$\mathbf {0.930\pm 0.064}$	$0.775\pm 0.070$	0.3 / 13.9 / 85.8
	$\alpha =6$	$0.858\pm 0.069$	$0.907\pm 0.079$	$0.785\pm 0.070$	$\mathbf {0.3}$ / $\mathbf {10.7}$ / $\mathbf {89.0}$
	$\alpha =8$	$\mathbf {0.886\pm 0.059}$	$0.879\pm 0.091$	$\mathbf {0.786\pm 0.077}$	0.3 / 12.1 / 87.7
	Average	$0.854\pm 0.069$	$0.911\pm 0.076$	$0.785\pm 0.069$	0.3 / 11.0 / 88.7

The best-performing individual network U-Net++, based on mean Intersection over Union (mIoU), served as a baseline for the case study. The last column shows the ratio of segmentations with low ($\text {mIoU} \le 0.5$), medium ($0.5< \text {mIoU} \le 0.7$), and high ($\text {mIoU} > 0.7$) quality. Significant values are in bold.

**Fig. 2:**
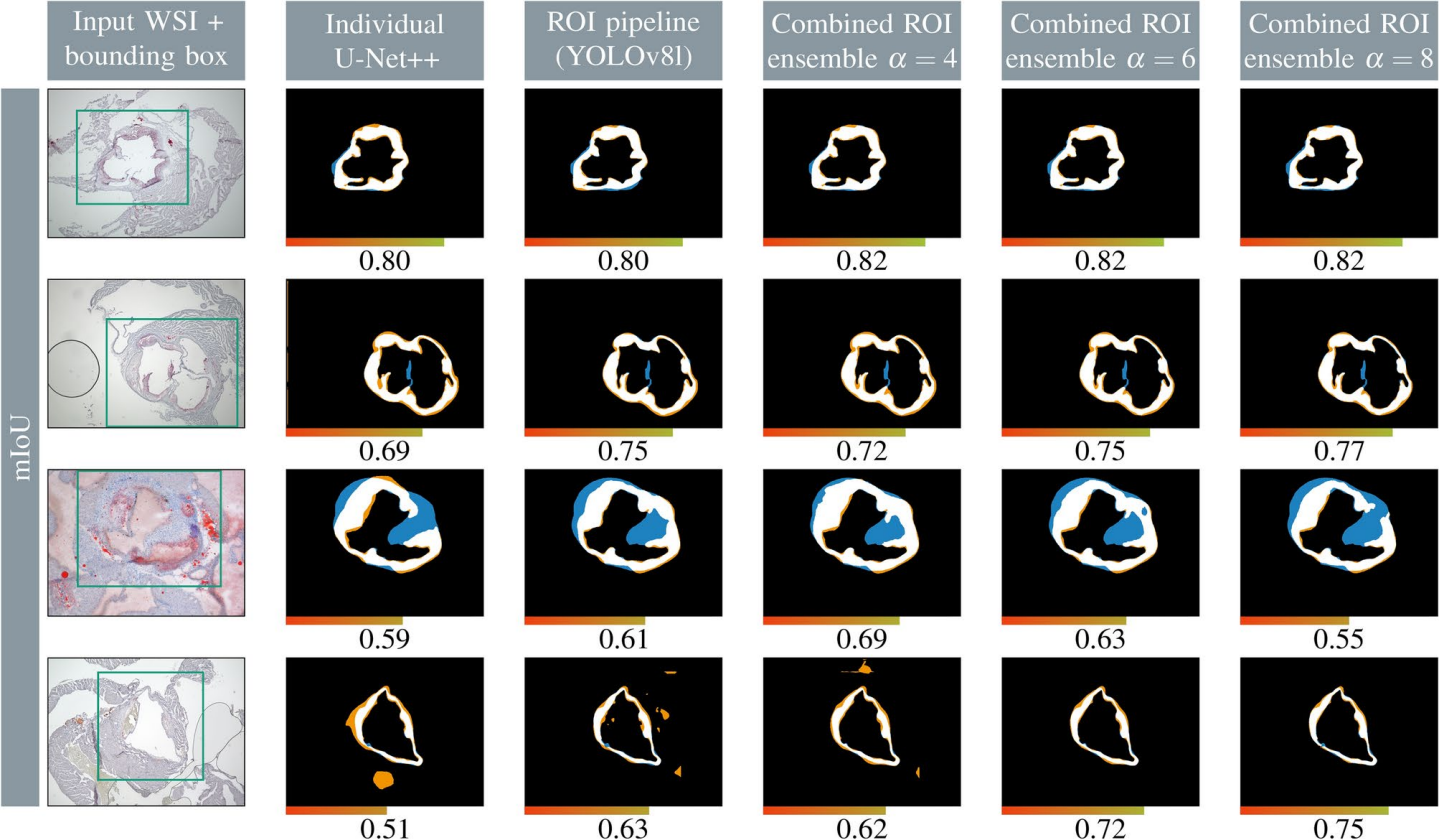
Qualitative examples of the individual network, the ROI pipeline, as well as the ensemble with different $\alpha$ overlapping parameters. TP = white, FP = orange, FN = blue, TN = black. GT = white + blue, prediction = white + orange. The mIoU is depicted below each sample along with a color bar. The green box in the input image shows the region of interest (ROI) used by the ROI pipeline.

To access the visual quality of the different methods, Fig. 2 shows output masks of the baseline U-Net++, the ROI pipeline, and the combined ROI ensemble. For the combined ROI ensemble, three different $\alpha$ hyperparameters for the overlapping combination strategy are displayed. The examples also depict TP, FP, FN, and TN pixels with different colors to visualize the over- or under-segmented areas. A broad range of WSIs is depicted, where the individual U-Net++ achieved a mIoU in the low to high range, indicating the potential for performance improvement. If the U-Net++ baseline reached a high mIoU ($\sim 0.8$), neither the ROI pre-processing pipeline nor the combined ROI ensemble significantly increased segmentation accuracy. However, the combined ROI ensemble strategy notably improved artery segmentation in difficult cases where the mIoU was low when using only a single network. Overall, the best performance was achieved when using a combined ROI ensemble strategy with $\alpha =6$, resulting in higher precision and fewer artifacts.

### Plaque segmentation

**Fig. 3:**
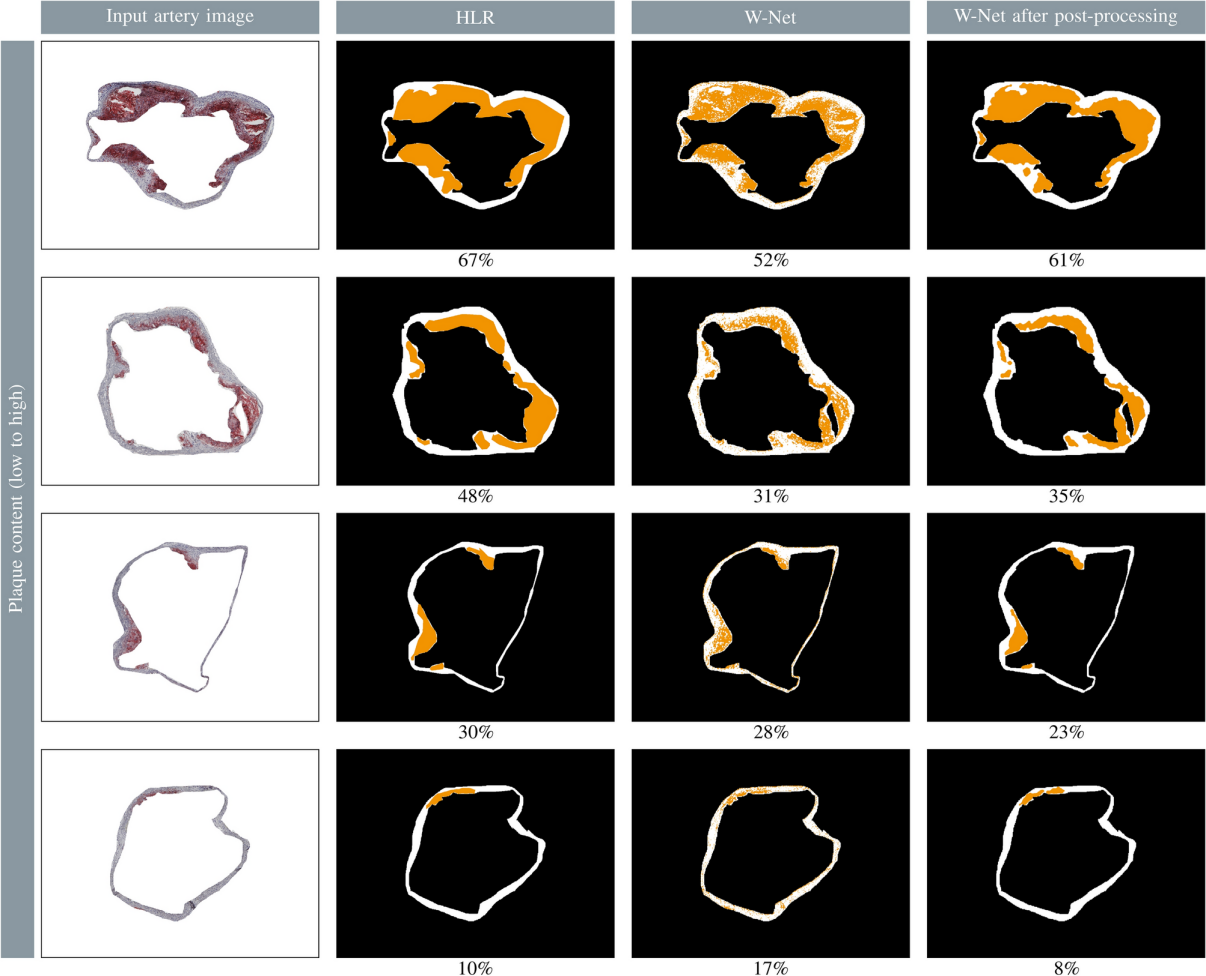
Examples showing plaque contents (detected plaque shown in orange, arterial region in white) according to hand-labeled reference (HLR), W-Net output mask, and W-Net output mask after post-processing. The plaque amount is depicted below each sample as a percentage of the artery mask.

After artery segmentation, plaques must be segmented inside the arterial region. To achieve this, an unsupervised W-Net method was trained, followed by subsequent post-processing. To access the visual appearance of the plaques, qualitative examples are illustrated in Fig. 3, showing input images, the corresponding hand-labeled references (HLR), W-Net output masks, and output masks after post-processing.

While the W-Net mask was pixel-based, the post-processed mask provided information about individual clusters, which coincided well with HLR masks. However, very small plaque clusters were often missing in the post-processed W-Net masks. Overall, the post-processed W-Net plaque areas appeared systematically smaller than the HLR. Notably, manual segmentation may not accurately capture the fragmented plaque structures, which are characterized by additional dents and gaps. The sizes of estimated plaques by W-Net and post-processing exhibited a strong correlation with HLR masks (Pearson’s $\text {r}=0.94$). Out of 1089 WSIs, 349 WSIs were identified as empty, without apparent plaques inside the arterial wall. Approximately $92\%$ of those were correctly detected as empty by the post-processed W-Net, while the remaining $8\%$ WSIs were predicted to have an average arterial plaque content of $\sim 3\%$. The remaining 740 WSIs had varying plaque contents up to a maximum of $77\%$. Among these, $8.2\%$ were incorrectly predicted to be empty due to the small area of plaques (an average of $4\%$ plaque content). For the remaining non-empty WSIs ($91.8\%$), a precision of $0.94\pm 0.09$ and recall of $0.54\pm 0.2$ were achieved.

### Case study

After optimization, we applied our two segmentation methods (artery and plaque) to a peer-reviewed mouse study^9^, where ORO staining had been manually assessed for mice subjected to a chow diet or high caloric western type diet (WTD).

Compared to the baseline network U-Net++, both the averaging combination strategy and the combined ROI ensemble demonstrated superior performance (see Table 1, Case study). Most importantly, the number of high-quality masks with mIoU scores above 0.7 increased by $\sim 8\%$. However, the effectiveness of the overlapping combination strategy varied with the $\alpha$ hyperparameter value, with $\alpha =6$ yielding the best results.

**Fig. 4:**
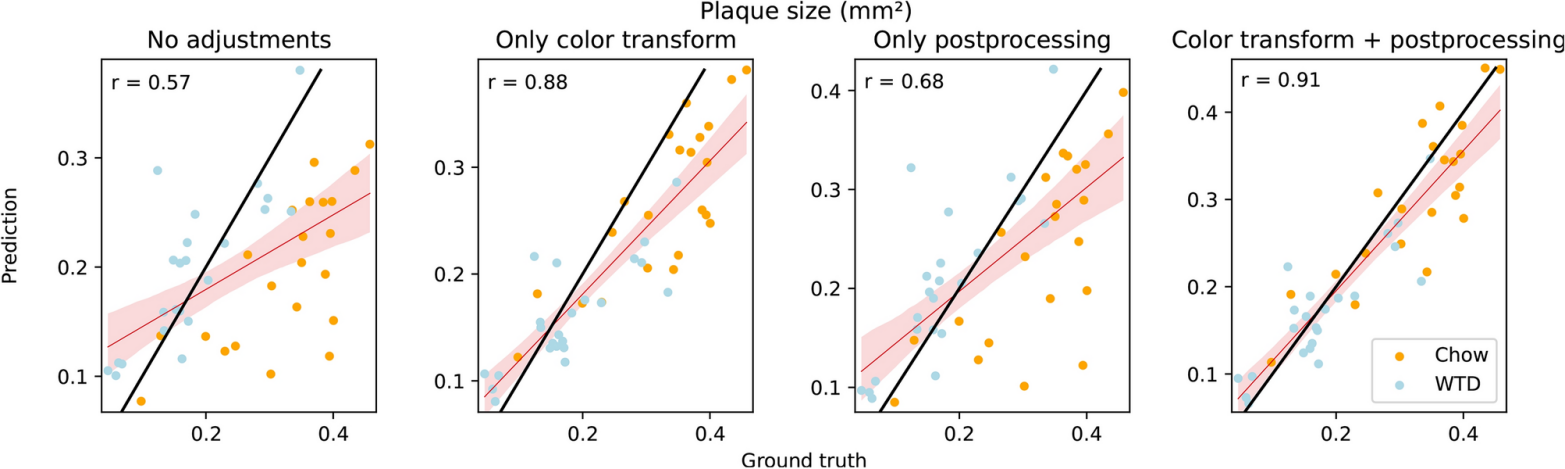
Linear regression of predicted plaque sizes (in $\hbox {mm}^2$) by the W-Net, with and without post-processing and color transfer, compared to the threshold-based reference (TBR) per mouse under chow or western type diet (WTD) from published reference data^9^. The Pearson correlation coefficient r is depicted in the top left corner. Black lines resemble perfect linear regression.

In the second stage, the AI-based plaque segmentation was evaluated using the previously predicted artery masks with the combined ROI ensemble with $\alpha =6$. Due to the inhomogeneous color spaces in the case study dataset, color transfer was applied to the input images before W-Net inference. Figure 4 illustrates the improvements in linear regression fits for the predicted plaque sizes (in $\hbox {mm}^2$ per mouse) compared to the reference plaque size when color transfer and post-processing were applied. The Pearson correlation coefficient increased from $\text {r}=0.57$ without adjustment to $\text {r}=0.91$ with these modifications.

**Fig. 5:**
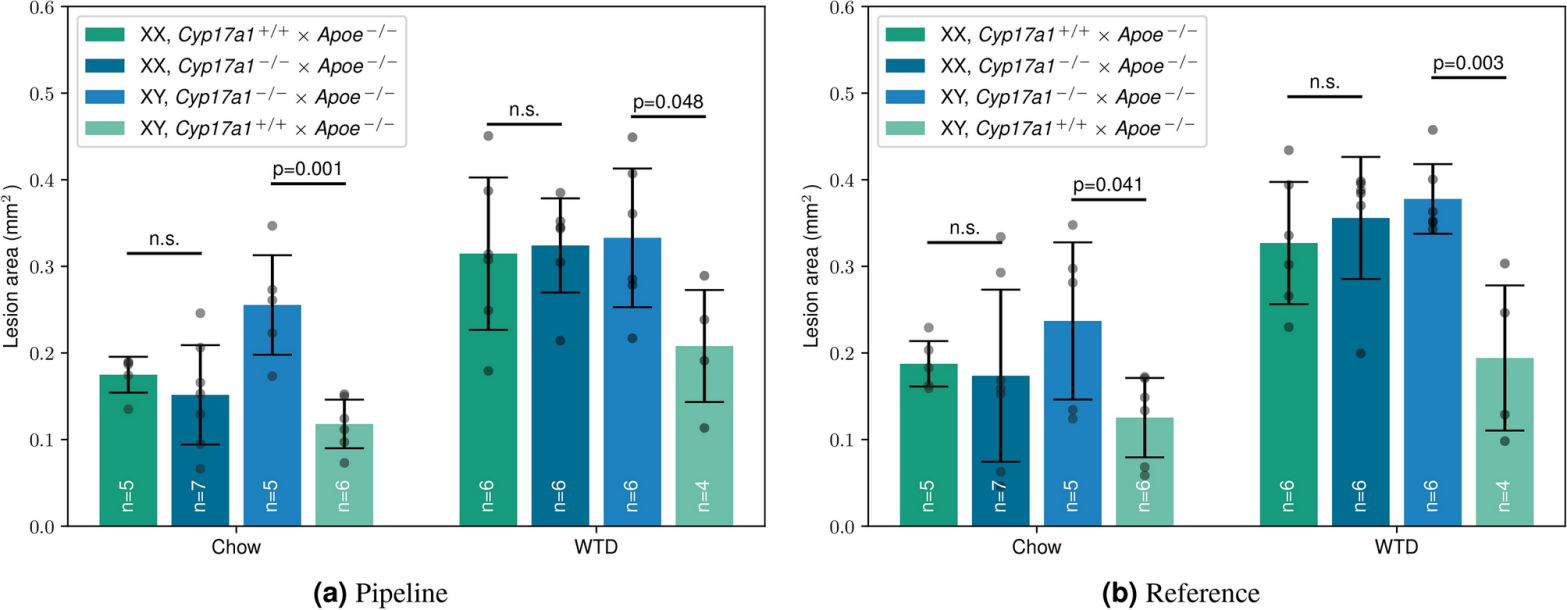
Comparison of our AI-based segmentation pipeline with published reference data^9^. The data points represent the medians per mouse for lesion areas of individual WSIs. The student’s t-test was used to calculate p-values between groups.* n.s.* not significant,* WTD* western type diet,* chow* control diet.

To assess whether our AI-based WSI segmentation can reproduce the biological findings reported by Aherrahrou et al.^9^, the plaque sizes are displayed by sex, genotype, and diet in Fig. 5. Overall, the results align closely, with only minor deviations in the group distributions. The W-Net-derived plaques were slightly smaller in the WTD group compared to the reference values. Nevertheless, the data support the conclusion that *Cyp17a1* promotes atherosclerosis in male mice, but not in female mice, regardless of diet type, which aligns with observations in human studies.

## Discussion

Animal models remain essential for understanding disease-driving mechanisms in complex diseases such as atherosclerosis. A search in Google Scholar for the terms “atherosclerosis”, “mice”, and “Oil Red O” yields about 20.000 articles published between 2013 and 2023. Therefore, there is still a great need for automatic analysis of atherosclerotic plaques. In this study, we present a pipeline that uses machine learning to segment cross-sections of the aortic root in transgenic mice and quantify atherosclerotic plaques. Our findings show that supervised machine learning approaches, using manually selected masks as ground truth, are suitable for arterial segmentation, with a single network (U-Net++) already providing a robust baseline. U-Net models^23^ have previously been applied to similar tasks, such as the segmentation of H&E-stained WSIs of brachiocephalic arteries into the lumen, plaque, and background by Murray et al.^14^. However, arterial segmentation of ORO-stained aortic roots is even more complex and requires a large training set and additional optimizations. To address this challenge, we implemented a two-step approach: first, we used the YOLOv8l framework to identify the region of interest (ROI), thereby excluding surrounding tissue. Second, we utilized ensembles of neural networks to improve the segmentation performance. While the ROI pipeline is faster, it is more prone to errors if the input bounding box is incorrect, potentially leading to cumulative errors. In contrast, the ensemble requires ten times the inference time of an individual network but offers improved robustness. Furthermore, the ROI method requires images with a high resolution, which was not the case for all WSIs. The resolution of the WSI must be sufficiently high, ensuring that the cropped ROI exceeds the network’s input size. Otherwise, it may contain less information than the unaltered image. Overall, the segmentation performance is similar for the individual methods (ROI pipeline or snapshot ensemble with averaging or overlapping) and is only improved by their combination. As a sum of both methods, the combined ROI ensemble pipeline effectively balances the close-up details of arterial structures with the broader context of the full-scale WSI for localization. This approach is similar to HookNet^15^, proposed by van Rijthoven et al., which employs two networks trained on different WSI resolutions. The feature spaces from both networks are then concatenated to integrate context and detail. In our method, the entire artery is used as the ROI instead of high-detail concentric patches, reducing dependence on correct patch size parameters or artery position. In addition, the cumulative error from an incorrect input bounding box can be mitigated, making this combined method more robust. Our analyses also revealed that the $\alpha$ hyperparameter of the overlapping strategy functions as a trade-off between FP and FN predicted pixels. This allows the output mask to be manually adjusted, for example with a $\alpha$-slider in a later-on application. The qualitative examples in Fig. 2 also illustrate this FP / FN trade-off, demonstrating that the optimal $\alpha$ may vary for each image. Depending on the $\alpha$ value, some gaps can be closed while noise can be removed. Thus, the ensemble output is more flexible compared to methods such as averaging, majority voting, or Bayesian voting, which have been widely applied for segmentation tasks^12,17–19^. This highlights an advantage of the overlapping strategy and its intuitive applicability. With this setup, high-quality artery masks can be created in seconds instead of several minutes. Furthermore, the different consensus phases can be visualized and presented as an uncertainty estimation.

In the second step of quantifying atherosclerotic lesions, plaque areas must be identified within the arterial wall. We used the unsupervised W-Net^37^ architecture for this purpose. In combination with post-processing steps, including morphological operations and pixel clustering, the prediction of plaque areas achieved high accuracy $\text {precision} = 0.94$ and a strong correlation with manually selected plaque masks ($\text {Pearson's r}>0.9$). This correlation could be even higher with increased accuracy in manual plaque area selection, as the complex shape of the interface between the plaque and the arterial wall has not been captured perfectly due to individual bias. While the prediction tended to underestimate the plaque content, manual segmentation often overestimated the plaque area due to missing fine-grained details, which is reflected by a high number of FN ($\text {recall} = 0.54$). Lastly, some small plaque clusters were missing in the post-processed W-Net mask, likely due to the applied thresholding to remove the noise. For WSIs with no plaque, the majority were correctly predicted as such, with the remaining outliers predicted to have very low plaque content near zero. These outliers are unlikely to significantly impact overall results and their biological interpretation. Nevertheless, an adaptive threshold could be employed to remove plaque clusters based on the total amount of predicted plaque. For example, the threshold could be set lower for masks with an overall low amount of predicted plaque before post-processing to remove noise more efficiently. In comparison, Fouad et al.^21^ proposed another unsupervised method solely using morphological operations and color analysis for H&E-stained cancer tissue WSIs. One downside of their approach is the requirement for hand-selected features, which introduce human bias to their method. A CNN-based approach using a clustering method was proposed by Faust et al.^20^, where H&E-stained WSIs were divided into patches and passed through a VGG19 CNN for feature extraction and clustering. Each cluster provided a class assignment for each patch. One limitation of their approach is the requirement of very high-resolution WSIs, as the used patch size is $1024\times 1024$ pixels. For our application, similar methods would only yield a coarse segmentation mask of the plaque.

Finally, we evaluated our optimized three-step segmentation pipeline using published reference data from Aherrahrou et al.^9^. For artery segmentation, the combined ROI ensemble consistently outperformed the baseline U-Net++ across all metrics, although the overall performance was lower compared to the original training dataset. This reduction in accuracy may be due to the lower quality of input WSIs in the reference study, likely resulting from older microscopy equipment or different acquisition settings. Under these circumstances, the advantage of the combined ROI ensemble becomes particularly evident: the poorer the performance of individual networks, the greater the improvement through their combination. Regarding the plaque segmentation, further refinement was achieved by applying a color transfer to correct inhomogeneities and W-Net post-processing steps to reduce artifacts. Adjusting the color space rendered the artery tissue more uniform, making the plaques more distinct from the surrounding tissue structures for the W-Net. Other color transfer methods could also be investigated in the future. To further improve the automated identification of plaque areas, a large dataset of WSIs with fully labeled plaques would be beneficial for supervised training. Such a training set could be generated by combining color-space segmentation (such as TBR), W-Net segmentation, and manual fine-tuning of polygon-based annotations.

Lastly, small deviations between the pipeline analysis and the reference hand-made analysis remained (Fig. 5), likely due to hand-made errors and/or cumulative errors within the pipeline. Therefore, it is still crucial to have an adequate number of mice in the study to effectively detect changes in atherosclerotic burden. Despite these discrepancies, the case study’s key findings remained consistent, demonstrating significant differences between the respective mice groups. This underscores the usage of the proposed pipeline, which can analyze multiple hundreds of WSIs in mere seconds while providing the same insights into atherosclerotic development as manual analysis. However, while a fully automated system would be ideal, it is not yet feasible in the medical field, as manual verification is always required. Therefore, it is important to minimize the proportion of remaining manual work as much as possible.

## Conclusion

We are confident that AI-based automated analyses of atherosclerotic plaques will significantly accelerate cardiovascular research and the development of novel therapies worldwide. Therefore, we propose a fully automatic pipeline for lesion quantification in murine aortic roots, combining supervised and unsupervised methods for artery and plaque segmentation, respectively. While an individual network can provide adequate artery segmentation in most cases, difficult examples often require manual corrections. In such instances, an ensemble of networks is recommended to improve arterial segmentation and reduce the number of low-quality outputs, despite enhanced processing time. Furthermore, our newly proposed strategy of overlaying segmentation masks from different neural networks, in conjunction with a variable hyperparameter $\alpha$, has demonstrated superior performance with minimal human intervention. This strategy has two key advantages: the artery prediction is more adaptable and overlapping masks can visualize the certainty of the output prediction as a form of explainable AI. Regarding plaque segmentation, the adjusted W-Net provides suitable plaque predictions, which can be further improved through post-processing or, in the case of an inhomogeneous dataset, by applying color transfer. The combination of best-performing artery and plaque segmentation strategies yielded results comparable to hand-made plaque analysis from a peer-reviewed mouse study, leading to the same biological conclusions. This example demonstrates that our pipeline can help accelerate atherosclerosis research and provide further insights into this complex cardiovascular disease.

## Data Availability

The used datasets and code are not publicly available due to further ongoing research. However, access may be granted to selected researchers upon reasonable request. If inquired, please contact johann.christopher.engster@imte.fraunhofer.de and zouhair.aherrahrou@uni-luebeck.de.
